# Novel Associations Between Mid-Pregnancy Cardiovascular Biomarkers and Preeclampsia: An Explorative Nested Case-Control Study

**DOI:** 10.1007/s43032-023-01445-z

**Published:** 2024-01-22

**Authors:** Paliz Nordlöf Callbo, Katja Junus, Katja Gabrysch, Lina Bergman, Inger Sundström Poromaa, Susanne Lager, Anna-Karin Wikström

**Affiliations:** 1 Department of Women’s and Children’s Health, Uppsala University, Akademiska sjukhuset, SE 751 85 Uppsala, Sweden; 2 Uppsala Clinical Research Centre, Uppsala, Sweden; 3 Department of Obstetrics and Gynecology, Institute of Clinical Sciences, Sahlgrenska Academy, University of Gothenburg, Gothenburg, Sweden; 4 Department of Obstetrics and Gynecology, Stellenbosch University, Cape Town, South Africa

**Keywords:** Preeclampsia, Matrix metalloproteinase-12, Natriuretic peptide B, Alpha-L-iduronidase, Boruta algorithm, Predictive biomarkers

## Abstract

Prediction of women at high risk of preeclampsia is important for prevention and increased surveillance of the disease. Current prediction models need improvement, particularly with regard to late-onset preeclampsia. Preeclampsia shares pathophysiological entities with cardiovascular disease; thus, cardiovascular biomarkers may contribute to improving prediction models. In this nested case-control study, we explored the predictive importance of mid-pregnancy cardiovascular biomarkers for subsequent preeclampsia. We included healthy women with singleton pregnancies who had donated blood in mid-pregnancy (~ 18 weeks’ gestation). Cases were women with subsequent preeclampsia (*n* = 296, 10% of whom had early-onset preeclampsia [< 34 weeks]). Controls were women who had healthy pregnancies (*n* = 333). We collected data on maternal, pregnancy, and infant characteristics from medical records. We used the Olink cardiovascular II panel immunoassay to measure 92 biomarkers in the mid-pregnancy plasma samples. The Boruta algorithm was used to determine the predictive importance of the investigated biomarkers and first-trimester pregnancy characteristics for the development of preeclampsia. The following biomarkers had confirmed associations with early-onset preeclampsia (in descending order of importance): placental growth factor (PlGF), matrix metalloproteinase (MMP-12), lectin-like oxidized LDL receptor 1, carcinoembryonic antigen-related cell adhesion molecule 8, serine protease 27, pro-interleukin-16, and poly (ADP-ribose) polymerase 1. The biomarkers that were associated with late-onset preeclampsia were BNP, MMP-12, alpha-L-iduronidase (IDUA), PlGF, low-affinity immunoglobulin gamma Fc region receptor II-b, and T cell surface glycoprotein. Our results suggest that MMP-12 is a promising novel preeclampsia biomarker. Moreover, BNP and IDUA may be of value in enhancing prediction of late-onset preeclampsia.

**Supplementary Information:**

The online version contains supplementary material available at 10.1007/s43032-023-01445-z.

## Introduction

Preeclampsia complicates 2–8% of pregnancies worldwide, the health burden being greatest in low- and middle-income settings [1, 2]. This multifaceted syndrome is one of the leading causes of maternal and neonatal morbidity and mortality [3]. In addition, women who have experienced preeclampsia run a double risk for cardiovascular incidents later in life [4].

Preeclampsia is defined by de novo onset of hypertension after 20 weeks’ gestation combined with signs of involvement of other organs, e.g., proteinuria. Although the pathophysiology of preeclampsia remains elusive, several underlying mechanisms have been identified, including abnormal placentation, oxidative stress, vascular inflammation, and impaired cardiovascular function [5, 6]. Preeclampsia may be divided into the subtypes early- and late-onset preeclampsia, defined as preeclampsia diagnosed before or from 34 weeks’ gestation [7]. These subtypes are suggested to develop via partly disparate pathological routes, eventually converging in common clinical symptoms. Compared to late-onset, early-onset preeclampsia has a stronger association with abnormal placentation, adverse cardiovascular changes, and future maternal cardiovascular outcomes [8–10].

Early detection of women at high risk for preeclampsia is essential for initiation of aspirin prophylaxis and improvement of outcomes [11]. Various preeclampsia prediction models have been developed, the result depending on the variables included, gestational week at assessment, and the targeted subtype [10, 11]. The models of greatest predictive performance are complex and include biomarkers, most commonly the angiogenic biomarker placental growth factor (PlGF) and its antagonist soluble fms-like tyrosine kinase (sFlt-1) [12–15]. However, in these prediction models, which usually include first-trimester multivariable parameters, the detection rate of overall preeclampsia is limited to around 54% (10% false-positive rate) [16]. Nevertheless, they may detect 90% of women at risk of early-onset preeclampsia, while the prediction rate for late-onset and post-term (delivery ≥ 37 gestational weeks) preeclampsia remains substantially lower [17–19]. Late-onset preeclampsia is, however, more common and may present with severe features, and thus, further exploration of potentially predictive biomarkers is warranted.

Preeclampsia and cardiovascular disease share pathophysiological entities and risk factors, making cardiovascular predictive biomarkers for preeclampsia an interesting topic for investigation. Cardiovascular biomarkers have to some extent been explored in pregnant women, both before clinical symptoms and at the time of manifest preeclampsia [20–22]. In longitudinal studies, early- and late-onset preeclampsia have shown a distinct cardiovascular profile before and throughout pregnancy, e.g., differences in cardiac output and vascular resistance to name a few [23]. We therefore hypothesize that early- and late-onset preeclampsia may be associated with different cardiovascular biomarkers before the onset of the disease.

Due to the complex nature of preeclampsia, machine learning algorithms, trained by large data sets to recognize and predict complex patterns, may be applied when exploring the predictive performance of multiple biomarkers [24, 25]. Therefore, we explored the individual predictive value of 92 cardiovascular biomarkers in mid-pregnancy plasma to detect subsequent preeclampsia using proteomic profiling and machine learning. Early-and late-onset preeclampsia were explored separately.

## Methods

### Study Population

This was an explorative nested case-control study. The source population was pregnant women from the population-based Uppsala University Hospital Biobank of Pregnant Women [26]. Women aged 18 or older attending their early second-trimester routine ultrasound scan at the University Hospital in Uppsala were invited to donate a blood sample for the biobank. If they accepted, a venous blood sample was collected in ethylenediaminetetraacetic acid-containing tubes. The samples were centrifuged (1500 g for 10 min) and stored at − 70 °C within 2 h after sampling. During the study period (2007–2018), 40% of the pregnant population in Uppsala County donated blood samples to the biobank (*n* = 15,000) [26]. The participating women gave written consent, and the Regional Ethical Review Board in Uppsala, Sweden, approved the study (Dnr 2007/181, Dnr 2018/251).

From the biobank, we included women with singleton pregnancies who had donated a blood sample between 16 + 0 and and 20 + 6 weeks’ gestation and had given birth at 22 weeks’ gestation or later. To ensure homogeneity, women with chronic hypertension, pre-gestational or gestational diabetes, known renal disease, and ongoing treatment with immune- or coagulation-modulating medication (including aspirin) or lithium were not eligible for inclusion. In Sweden, the use of aspirin for preeclampsia prevention was relatively rare during the study period, approximately 1–2% of nulliparous women receiving treatment [11], and women with a history of late-onset preeclampsia were usually not offered aspirin prophylaxis.

Cases were women with subsequent preeclampsia. The cases were categorized into early- and late-onset, defined as preeclampsia diagnosed before or ≥ 34 weeks’ gestation. Additionally, we studied term-onset preeclampsia separately, defined as preeclampsia diagnosed ≥ 37 weeks’ gestation. A diagnosis of preeclampsia was identified by corresponding International Classification of Diseases (ICD) codes (O14, O15), reported by the responsible physician at post-delivery discharge. If a woman participated in the biobank with repeated pregnancies complicated by preeclampsia, the pregnancy where the woman had the earliest onset of preeclampsia was included. During the study period, preeclampsia was clinically defined as new-onset of hypertension (systolic blood pressure ≥ 140 mmHg and/or diastolic blood pressure ≥ 90 mmHg) measured on two subsequent occasions at least 4 h a part together with proteinuria (≥ 300 mg/24 h or a spot urine protein/creatinine ratio ≥ 30 mg/mmol or at least 1 g/L [2 +] on a dipstick test) after 20 weeks’ gestation [27].

Controls were healthy pregnant women without a history of preeclampsia who continued a normal pregnancy after blood sampling. Normal pregnancy was defined as a pregnancy without hypertension, isolated proteinuria, cholestasis of pregnancy, isoimmunization, maternal thromboembolism, oligo- or polyhydramnios, placental abruption, preterm delivery (before 37 weeks’ gestation), or delivery of an infant born small for gestational age or stillborn. Cases and controls were initially matched one-to-one based on parity and first-trimester body mass index (BMI). Maternal age, first-trimester smoking habits, gestational week at blood sampling, and storage time of blood samples in freezers were matched on a group level. After reviewing medical records for data collected on maternal, pregnancy, and infant characteristics and validation of preeclampsia diagnosis, some samples were excluded. The final cohort consisted of *n* = 296 preeclampsia cases and *n* = 333 controls.

### Biochemical Analyses

Plasma samples were analyzed at the Science for Life Laboratory, Uppsala, Sweden, using Olink’s Proseek multiplex Cardiovascular II (CVD-II) Panel containing 92 known cardiovascular and inflammatory markers. The analytical details of the proximity extension assay (PEA) technology and the full names of the biomarkers are presented in Supplementary Information and Supplemental Table 1 (S1). Based on negative controls included in the analyses, a limit of detection (LOD) was estimated for each PEA measurement. We included all proteins with the actual measured level (even when below LOD) for each sample instead of imputing values below LOD.

The data were reported as normalized protein expression (NPX) on a Log2-scale, i.e., relative protein values, where a one-unit increase in NPX corresponds to a doubling of the protein concentration [28].

### Statistical Analyses

Baseline characteristics are presented with mean and standard deviation for continuous variables and frequencies for categorical variables. In cases and controls, continuous variables were compared with the independent *t*-test and discrete variables with Pearson’s chi-squared test.

In the main analyses, we applied a machine learning approach, the Boruta algorithm. This method is built on random survival forest (RF), which determines an unbiased grading of the predictive importance of all variables, i.e., Olink biomarkers PEA levels and clinical characteristics (parity, maternal age, first-trimester BMI, smoking habits, systolic and diastolic blood pressure, and gestational age at blood sampling) [29]. The Boruta algorithm is than applied to select variables that have a variable importance higher than a random association with preeclampsia development. The Boruta algorithm performs multiple RF runs with added random variables (i.e., shuffled copies of the original variables) to already existing variables and iteratively compares the importance of the original variables with the added random variables and classifies them. Variables performing better or worse than the random noise are classified as confirmed or rejected. If not confirmed or rejected, the Boruta algorithm is considered to be indecisive and variables are classified as tentative. This approach captures all relevant variables instead of only non-redundant variables; thus, correlated variables may be included. Further, the Boruta algorithm identifies both linear and non-linear associations between a biomarker and an outcome [30, 31]. We performed separate analyses for overall preeclampsia, early- and late-onset preeclampsia, and preeclampsia with onset at term (≥ 37 gestational weeks).

We then applied logistic regression analyses to estimate the direction of the individual association between each of the 92 biomarkers and preeclampsia and its subtypes, early- and late-onset preeclampsia. We identified the following confounders by drawing and analyzing a directed acyclic graph: parity, maternal age, first-trimester BMI, first-trimester smoking habits, first-trimester systolic and diastolic blood pressure, and gestational age at blood sampling (Supplemental Fig. 1). Estimates were presented as adjusted odds ratios (aORs) with 95% confidence intervals (CIs), and Bonferroni’s *post hoc* test was applied to correct for multiple testing (92 proteins), and *p* values under 0.05/92 were considered significant. To determine the discriminative ability of the model, a receiver operating characteristics (ROC) curve was constructed, and the area under the curve (AUC) value was calculated.

The Boruta and logistic regression analyses were run on complete data sets. Missing values were few (all together: 5 blood pressures, 1 birth weight, 1 value for proheparin-binding epidermal growth factor-like growth factor) and were imputed using a chained equations approach with the predictive mean matching method [32].

Statistical analyses were done with R version 4.0.2 and the Statistical Package for the Social Science (SPSS) Statistics 27.0.

## Results

### Study Population

The clinical characteristics of the study population are presented in Table 1. Women with subsequent preeclampsia did not differ from the controls with regard to age, BMI, parity, and smoking habits. However, first-trimester systolic and diastolic blood pressures were higher in women with subsequent preeclampsia. Of the multiparous women with subsequent preeclampsia, 27 (25%) had a previous pregnancy complicated by preeclampsia. Compared to controls, women with subsequent preeclampsia had pregnancies of shorter duration and gave birth to infants with lower birth weights.

**Table 1:** Characteristics of the study population and preeclampsia cases (*n* = 296)

Characteristics	*n*	Healthy pregnancy *n* = 333	Preeclampsia *n* = 296	*p* value
Gestational length at sampling (weeks)	629	18.3 (± 0.8)	18.4 (± 0.9)	0.22
Maternal characteristics at first antenatal visit				
Age (years)	629	30.1 (± 4.7)	29.9 (± 5.1)	0.59
Body Mass Index (kg/m^2^)	629	26.4 (± 5.4)	26.5 (± 5.4)	0.76
Nulliparity	629	200 (60.1)	189 (63.9)	0.42
Smoker	629	12 (3.6)	7 (2.4)	0.37
Systolic blood pressure (mm Hg)	627	116 (± 10)	120 (± 11)	< 0.001
Diastolic blood pressure (mm Hg)	626	71 (± 8)	73 (± 9)	0.002
History of preeclampsia	629	0 (0%)	27 (9.1)	
Infant characteristics				
Gestational length at birth (weeks)	629	40.1 (± 1.1)	38.6 (± 1.8)	< 0.001
Birth weight (grams)	628	3682 (± 471)	3232.8 (± 781)	< 0.001
Sex: Girl	629	152 (45.6%)	153 (51.7)	0.13
Preeclampsia cases				
Gestational length at diagnosis (weeks)		37.6 (± 3.0)		
Small for gestational age ^b^		20 (13.5)		
Early-onset (< 34 gestational weeks)		30 (10.1)		
Late-onset (≥ 34 gestational weeks)		266 (90.5)		
HELLP^a^ syndrome		13 (4.4)		
Eclampsia		2 (0.7)		
Systolic blood pressure (mm Hg)				
At diagnosis		149.6 (± 12.8)		
Highest		157.9 (± 17.4)		
Diastolic blood pressure (mm Hg)				
At diagnosis		96 (± 9)		
Highest		99 (± 9)		
Antihypertensive treatment		189 (63.9)		
Symptoms				
Severe headache		6 (2.0)		
Severe upper abdominal pain		9 (3.0)		
Visual disturbance		10 (3.4)		
Hyperreflexia		19 (6.4)		

Data given as mean (± standard deviation) or number (percentage). Data on maternal, pregnancy, and infant characteristics, registered by midwives and physicians, were collected from the individual electronic medical charts. ^a^ Hemolysis, elevated liver enzymes, and low platelets. ^b^ Infant birth weight <  − 2 standard deviations for the gestational age and sex

Among preeclampsia cases, the mean highest systolic blood pressure during pregnancy was 158 mmHg, and 189 women (64%) needed treatment with antihypertensive medication. We classified 30 women (10%) in the preeclampsia group as having early-onset preeclampsia (diagnosis < 34 weeks’ gestation).

### Variable Importance for Subsequent Development of Preeclampsia as Determined by Machine Learning

**Fig. 1:**
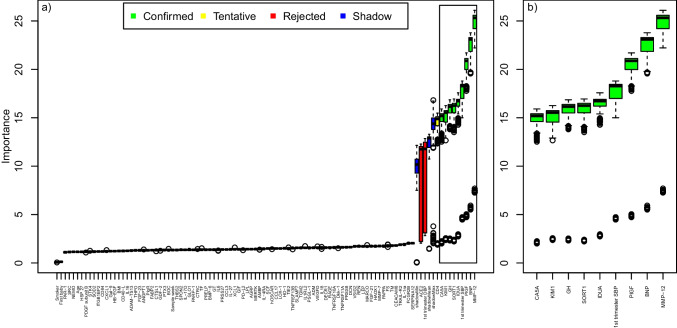
Variable importance for subsequent preeclampsia by machine learning (*n* cases = 296; *n* controls = 333. Matched on all maternal characteristics, except first trimester blood pressure). **a** Boruta analysis, listing the variable importance (y-axis) for subsequent preeclampsia for all variables (x-axis) including 92 biomarkers’ PEA measurements and maternal characteristics (parity, maternal age, BMI, smoking, first trimester systolic and diastolic blood pressure and gestational age at blood sampling). The black center line in the boxplots denotes the median value (50th percentile), while the colored box contains the 25th to 75th percentiles of the importance. The black whiskers mark the 5th and 95th percentiles, and values beyond these upper and lower bounds are considered outliers, marked with white dots. The green boxes represent the confirmed variables, performing better than the reference levels. The red boxes represent the rejected variables, performing worse than the reference levels. The yellow boxes represent tentative variables, performing neither better nor worse than the reference levels. The importance of randomized variables is shown in blue and represent the reference levels. **b** Variables with confirmed importance, in **a**, are enlarged and shown

When all biomarkers and maternal characteristics were included in the Boruta algorithm, nine variables were confirmed that had a larger than random association with overall risk of preeclampsia development. These, in descending order of the median importance, were matrix metalloproteinase (MMP)-12, natriuretic peptide B (BNP), PlGF, first-trimester systolic blood pressure, alpha-L-iduronidase (IDUA), sortilin, growth hormone, kidney injury molecule, and mitochondrial carbonic anhydrase 5A (Fig. 1).

**Fig. 2:**
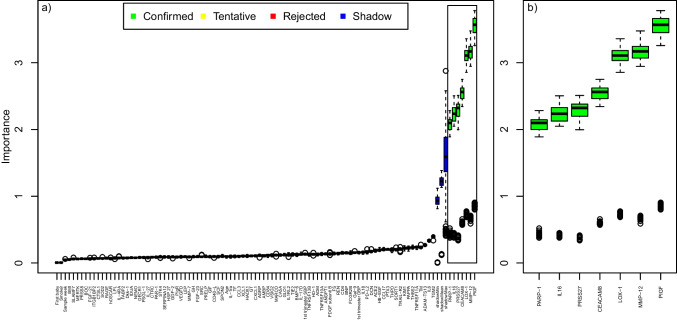
Variable importance for subsequent early-onset preeclampsia by machine learning (*n* cases = 30; *n* controls = 27. Matched on all maternal characteristics, except first trimester blood pressure). **a** Boruta analysis, listing the variable importance (y-axis) for subsequent early-onset preeclampsia for all variables (x-axis) including 92 biomarkers’ PEA measurements and maternal characteristics (parity, maternal age, BMI, smoking, first trimester systolic and diastolic blood pressure and gestational age at blood sampling). The black center line in the boxplots denotes the median value (50th percentile), while the colored box contains the 25th to 75th percentiles of the importance. The black whiskers mark the 5th and 95th percentiles, and values beyond these upper and lower bounds are considered outliers, marked with white dots. The green boxes represent the confirmed variables, performing better than the reference levels. The red boxes represent the rejected variables, performing worse than the reference levels. The yellow boxes represent tentative variables, performing neither better nor worse than the reference levels. The importance of randomized variables is shown in blue and represent the reference levels. **b** Variables with confirmed importance, in **a**, are enlarged and shown

**Fig. 3:**
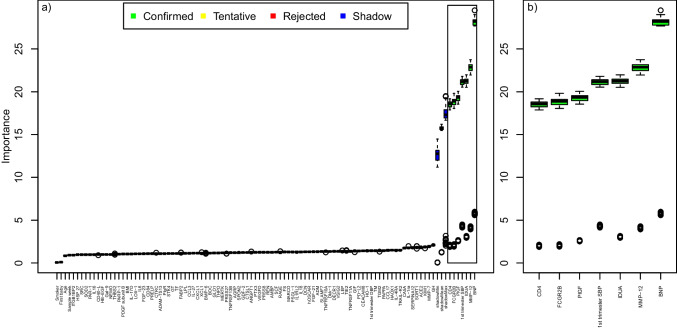
Variable importance for subsequent late-onset preeclampsia by machine learning (*n* cases = 266; *n* controls = 258. Matched on all maternal characteristics, except first trimester blood pressure). **a** Boruta analysis, listing the variable importance (y-axis) for subsequent late-onset preeclampsia for all variables (x-axis) including 92 biomarkers’ PEA measurements and maternal characteristics (parity, maternal age, BMI, smoking, first trimester systolic and diastolic blood pressure and gestational age at blood sampling). The black center line in the boxplots denotes the median value (50th percentile), while the colored box contains the 25th to 75th percentiles of the importance. The black whiskers mark the 5th and 95th percentiles, and values beyond these upper and lower bounds are considered outliers, marked with white dots. The green boxes represent the confirmed variables, performing better than the reference levels. The red boxes represent the rejected variables, performing worse than the reference levels. The yellow boxes represent tentative variables, performing neither better nor worse than the reference levels. The importance of randomized variables is shown in blue and represent the reference levels. **b** Variables with confirmed importance, in **a**, are enlarged and shown

The Boruta algorithm confirmed that seven variables were associated with early-onset preeclampsia. In descending order of their median importance, these were PlGF, MMP-12, lectin-like oxidized LDL receptor 1 (LOX-1), carcinoembryonic antigen-related cell adhesion molecule 8, serine protease 27, pro-interleukin-16, and poly (ADP-ribose) polymerase 1 (Fig. 2). Concerning late-onset preeclampsia, we found associations with the variables BNP, MMP-12, IDUA, first-trimester systolic blood pressure, PlGF, low-affinity immunoglobulin gamma Fc region receptor II-b (FCGR2B), and T cell surface glycoprotein (CD4), presented in descending order of their median importance (Fig. 3).

In a sub-analysis, the Boruta algorithm confirmed six variables with larger than random associations, with term onset of preeclampsia. These, in descending order of their median importance, were BNP, Serpin A12, IDUA, first-trimester systolic blood pressure, CD4, and FCGR2B (results not shown).

### The Association of Biomarkers with Subsequent Preeclampsia

Adjusted logistic regression models showed an increased risk for later development of preeclampsia with decreasing levels of the following three biomarkers: MMP-12 (aOR 0.63, 95% CI 0.48–0.82), BNP (aOR 0.57, 95% CI 0.43–0.74), and PlGF (aOR 0.52, 95% CI 0.38–0.71). This means that one-unit reduction of the biomarkers was associated with 37%, 43%, and 48% risk increase, respectively. For IDUA, one-unit elevation was associated with 100% increased risk for later development of preeclampsia (aOR 2.00, 95% CI 1.36–2.94). The AUC values for the adjusted model were MMP-12 0.67, BNP 0.66, and PlGF 0.66. Further, the sensitivities at 90% specificity for the adjusted model were MMP-120.25, BNP 0.26, PlGF 0.28, and IDUA 0.21.

In further analyses of the biomarkers’ associations with early- and late-onset preeclampsia, significant associations were only found for the latter. One unit reduction of BNP showed a 46% increased risk for development of late-onset preeclampsia (aORs with CIs: 0.54, 95% 0.40–0.73). For IDUA, one-unit elevation was associated with 120% increased risk for late-onset preeclampsia (2.19, 95% 1.44–3.35). The AUC values for the adjusted model were BNP 0.68 and IDUA 0.68. Further the sensitivities at 90% specificity for the adjusted model were AUC BNP 0.23 and IDUA 0.21.

## Discussion

Through proteomic profiling and machine learning, this explorative study investigated the individual association between 92 cardiovascular biomarkers and the subsequent development of preeclampsia. We identified 15 biomarkers with independent associations to overall preeclampsia or its subtypes, early- and late-onset preeclampsia. In particular, MMP-12 is highlighted as a promising predictive mid-pregnancy biomarker for subsequent preeclampsia. For late-onset preeclampsia, where there are no established predictive biomarkers, BNP and IDUA were the two strongest biomarkers.

This study adds information on the association between decreased MMP-12 levels in early mid-pregnancy and later development of preeclampsia. However, a recent study found lower levels of MMP-12 during gestational weeks 11–13 in women who later developed preeclampsia, than were found in those who continued to have a normotensive pregnancy [21]. Interestingly, they found an increase of MMP-12 between the first and second (22–24 weeks) trimester in women with subsequent preeclampsia. In contrast, the levels decreased in those who continued to have a normotensive pregnancy. Therefore, we suspect that our finding of an association between decreased MMP-12 in early mid-pregnancy and subsequent preeclampsia would have been even stronger if the blood samples had been collected in the first trimester. Moreover, our findings strengthen the previous results of Yakovleva et al. because their results were not adjusted for maternal characteristics and other biomarkers such as PlGF. Further, we could separate the outcomes early-and late-onset preeclampsia. Interestingly, elevated plasma levels of another member of the MMP family, MMP3, is a risk factor for cardiovascular disease, and elevated levels of this protein are also associated to early-onset preeclampsia at time of diagnosis [22]. The pathophysiological background for the association between MMP-12 and preeclampsia development is unknown. In pregnant women, matrix metalloproteinases (MMPs) are engaged in uterine artery remodeling, vasodilation, and modulation of inflammation, processes that all are more or less deranged in preeclampsia [33]. Altered levels of MMPs may affect systemic vasodilation and vascular remodeling in the placenta, subsequently leading to preeclampsia. However, further knowledge of the MMP-12 and other MMPs regarding pathophysiology and predictive accuracy in preeclampsia is needed.

BNP was the biomarker with the highest predictive importance for late-onset and term preeclampsia. On the contrary, BNP could not be confirmed as a biomarker with predictive importance for early-onset preeclampsia. Our findings agree with previous studies, one of them conducted by our research group using part of the same study cohort as the present study. In that study, low plasma N-terminal proBNP (NT-proBNP) levels in the first or second trimester were associated with term birth but not early-onset or preterm preeclampsia [34, 35]. Inversely, higher NT-proBNP levels in early pregnancy have been associated with a lower risk of hypertensive disease in pregnancy and a lower long-term risk for hypertension [36]. BNP is secreted from the cardiac ventricles in response to ventricular volume expansion and pressure overload [36, 37]. In healthy pregnancies, the cardiovascular system compensates for the changes in blood volume and BNP levels remain stable [38]. As hypothesized by Hausprung et al., lower NT-proBNP and BNP levels in early pregnancy may reflect impaired cardiovascular adaption to the pregnancy and impaired pre-pregnancy cardiovascular function [36, 38, 39]. Our findings support the concept of diverse pathophysiological routes and cardiovascular profiles in early-onset vs. late-onset preeclampsia and suggest that BNP is a strong predictive biomarker for late-onset preeclampsia.

Consistent with previous studies, PlGF had a higher prediction capacity for early (< 34 weeks)- than for late (≥ 34 weeks)-onset preeclampsia [40, 41]. PlGF is incorporated into the most commonly used prediction models, including the internationally validated competing risk model by the Fetal Medicine Foundation [19]. These multivariable models are mainly designed to detect early-onset preeclampsia [19].

Predicting late-onset preeclampsia is challenging, and published multivariate models have poor predictive accuracy [15, 17, 42]. In our study, several novel predictive biomarkers were associated with late-onset preeclampsia, including IDUA, FCGR2B, and CD4. Albeit AUC values for the adjusted regression model were not clearly discriminative for late-onset preeclampsia, incorporation of these biomarkers into multivariable prediction models may improve the prediction of late-onset preeclampsia. The added cost would have to be balanced against the benefits such as the potential of reducing severe complications to the disease.

The major strengths of this study are the population-based cohort design and the detailed information on the population collected before the development of preeclampsia. Further, the large sample size enabled the exploration of early- and late-onset preeclampsia separately. Another strength was the availability of plasma samples from mid-pregnancy, enabling the exploration of biomarkers before the disease became clinically manifest. The matched design between cases and controls and the exclusion of women with co-morbid diseases entail both strengths and limitations. We selected this design to minimize factors that could confound the origin of the cardiovascular biomarkers [43]. Further, since the study is explorative, we preferred the homogeneity between cases and controls instead of results with high generalizability. However, future studies should investigate the predictive importance of biomarkers in the first trimester in more heterogeneous study populations, together with additional parameters of maternal characteristics and the mean uterine artery pulsatility index. Moreover, the relative protein values and lack of validation of the biomarkers studied are limitations of our study. However, a previous study has shown an excellent correlation between the relative protein values obtained with a CVD Olink panel and absolute protein values obtained by immuno-chemiluminescence analysis of PlGF [22].

## Conclusion

In conclusion, the results of the present study of cardiovascular biomarkers and their individual importance for prediction of preeclampsia found MMP-12 to be the most promising candidate, but we also highlight BNP and IDUA as potential predictors for late-onset preeclampsia. Future studies should focus on absolute protein levels and incorporate these biomarkers into multivariate prediction models for preeclampsia to evaluate their predictive accuracy. Further investigation of their performance in multi-step prediction models for late-onset preeclampsia is also warranted.

### Supplementary Information

Below is the link to the electronic supplementary material.

Supplementary file1 (DOCX 89 KB)

## Data Availability

The data that support the findings of this study are available from the corresponding author upon reasonable request. However, access is restricted because of ongoing studies on the data set and limitations in the ethical approval (including patient informed consents).
