# Supplementary material for: Novel Associations Between Mid-Pregnancy Cardiovascular Biomarkers and Preeclampsia: An Explorative Nested Case-Control Study
Source: Reprod Sci. 2024 Jan 22;31(5):1391–400. doi: 10.1007/s43032-023-01445-z (PMC11090924; doi:10.1007/s43032-023-01445-z)
Supplement: Supplementary file 1 — Supplementary file1 (DOCX 89 KB) [file 43032_2023_1445_MOESM1_ESM.docx]

Supporting information

Article title

Novel Associations Between Mid-Pregnancy Cardiovascular Biomarkers and Preeclampsia: an Explorative Nested Case-Control Study

Journal name

Reproductive Sciences

Names of the authors and affiliations

Paliz Nordlöf Callbo, MD; Katja Junus, MD, PhD; Katja Gabrysch, PhD; Lina Bergman, MD, PhD; Inger Sundström Poromaa, MD, PhD; Susanne Lager, MSc, PhD; Anna-Karin Wikström, MD, PhD

Corresponding author

Paliz Nordlöf Callbo

Department of Women´s and Children´s Health, Uppsala University, Sweden

Akademiska sjukhuset, SE 751 85 Uppsala, Sweden

Telephone number: 0046700913986; e-mail: [Paliz.Nordlof_Callbo@kbh.uu.se](mailto:Paliz.Nordlof_Callbo@kbh.uu.se)

Expanded materials and methods

*Biochemical analyses*

The samples were centrifuged (1,500g for 10 min) and stored at -70°C within two hours after sampling. Proximity extension assay (PEA) technology followed by quantification using real-time PCR on the Fluidigm BioMark HD real-time PCR platform were used to measure relative protein levels [1]. The high through-put multiplex immunoassay supplied by Olink® Target 96 uses proximity extension assay to measure 92 proteins across 96 samples simultaneously using one microliter of plasma. The proximity extension assay allows a pair of antibodies to pair-wise bind to the target protein present in the sample. A new real-time polymerase chain reaction (PCR) target sequence is produced by a proximity-dependent DNA polymerization event. The resulting sequence is detected and quantified using standard PCR. The data are reported as normalized protein expression (NPX), calculated on normalized Ct values in GenEx software using Olink Wizard. Estimations of the relation between NPX and absolute values are presented at <http://www.olink.com>. On each assay plate, both cases and controls were included. Each plate also contained interplate controls (allowing for adjustments of any differences between runs) and negative controls. The limit of detection (LOD) is based on the background plus three standard deviations estimated from negative controls and calculated separately for each sample plate. All 92 proteins had levels above LOD in more than 50% of the samples (Table S2-4).


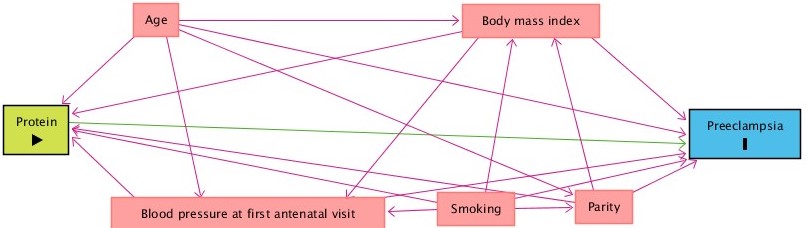


Fig. S1 Directed acyclic graph illustrating the causal effect of cardiovascular proteins on preeclampsia. Variables that could affect the relationship of the cardiovascular proteins (exposure) on preeclampsia (outcome) were identified as confounders: age, body mass index, blood pressure at the first antenatal visit, smoking, and parity. Colour coding of squares: yellow=exposure, blue=outcome, pink= confounders. Colour coding of arrows: green= casual path, purple=biasing path.

Table. S1 Biomarkers in Proseek Multiplex Cardiovascular II panel

| **Abbreviation** | **Full name** |
| --- | --- |
|  |  |
| ACE2 | Angiotensin-converting enzyme 2 |
| ADAM-TS13 | A disintegrin and metalloproteinase with thrombospondin motifs13 |
| ADM | Adrenomedullin |
| AGRP | Agouti-related protein |
| AMBP | Protein AMBP |
| ANGPT1 | Angiopoietin-1 |
| BMI | Body mass index |
| BMP-6 | Bone morphogenetic protein 6 |
| BNP | Natriuretic peptide B |
| BOC | Brother of CDO |
| CA5A | Carbonic anhydrase 5A, mitochondrial |
| CCL17 | C-C motif chemokine 17 |
| CCL3 | C-C motif chemokine 3 |
| CD4 | T-cell surface glycoprotein CD4 |
| CD40-L | CD40 ligand |
| CD84 | SLAM family member 5 |
| CEACAM8 | Carcinoembryonic antigenrelated cell adhesion molecule 8 |
| CTRC | Chymotrypsin C |
| CTSL1 | Cathepsin L1 |
| CXCL1 | C-X-C motif chemokine 1 |
| DCN | Decorin |
| DECR1 | 2,4-dienoyl-CoA reductase, mitochondrial |
| Dkk-1 | Dickkopf-related protein 1 |
| FABP2 | Fatty acid-binding protein, intestinal |
| FGF21 | Fibroblast growth factor 21 |
| FGF-23 | Fibroblast growth factor 23 |
| FS | Follistatin |
| Gal-9 | Galectin-9 |
| GDF-2 | Growth/differentiation factor 2 |
| GH | Growth hormone |
| GIF | Gastric intrinsic factor |
| GLO1 | Lactoylglutathione lyase |
| GT | Gastrotropin |
| HAOX1 | Hydroxyacid oxidase 1 |
| HB-EGF | Proheparin-binding EGF-like growth factor |
| HO-1 | Heme oxygenase 1 |
| hOSCAR | Osteoclast-associated immunoglobulin-like receptor |
| HSP 27 | Heat shock 27 kDa protein |
| IDUA | Alpha-L-iduronidase |
| IgG Fc receptor II-b | Low affinity immunoglobulin gamma Fc region receptor II-b |
| IL16 | Pro-interleukin-16 |
| IL-17D | Interleukin-17D |
| IL-18 | Interleukin-18 |
| IL-1ra | Interleukin-1 receptor antagonist protein |
| IL1RL2 | Interleukin-1 receptor-like 2 |
| IL-27 | Interleukin-27 |
| IL-4RA | Interleukin-4 receptor subunit alpha |
| IL6 | Interleukin-6 |
| ITGB1BP2 | Melusin |
| KIM1 | Kidney Injury Molecule |
| LEP | Leptin |
| LOX-1 | Lectin-like oxidized LDL receptor 1 |
| LPL | Lipoprotein lipase |
| MARCO | Macrophage receptor MARCO |
| MERTK | Tyrosine-protein kinase Mer |
| MMP-12 | Matrix metalloproteinase-12 |
| MMP-7 | Matrix metalloproteinase-7 |
| NEMO | NF-kappa-B essential modulator |
| PAPPA | Pappalysin-1 |
| PAR-1 | Proteinase-activated receptor 1 |
| PARP-1 | Poly [ADP-ribose] polymerase 1 |
| PDGF subunit B | Platelet-derived growth factor subunit B |
| PD-L2 | Programmed cell death 1 ligand 2 |
| PGF | Placenta growth factor |
| PIgR | Polymeric immunoglobulin receptor |
| PRELP | Prolargin |
| PRSS27 | Serine protease 27 |
| PRSS8 | Prostasin |
| PSGL-1 | P-selectin glycoprotein ligand 1 |
| PTX3 | Pentraxin-related protein PTX3 |
| RAGE | Receptor for advanced glycosylation end products |
| REN | Renin |
| SCF | Stem cell factor |
| SERPINA12 | Serpin A12 |
| SLAMF7 | SLAM family member 7 |
| SOD2 | Superoxide dismutase [Mn], mitochondrial |
| SORT1 | Sortilin |
| SPON2 | Spondin-2 |
| SRC | Proto-oncogene tyrosine-protein kinase Src |
| STK4 | Serine/threonine-protein kinase 4 |
| TF | Tissue factor |
| TGM2 | Protein-glutamine gamma-glutamyltransferase 2 |
| THBS2 | Thrombospondin-2 |
| THPO | Thrombopoietin |
| TIE2 | Angiopoietin-1 receptor |
| TM | Thrombomodulin |
| TNFRSF10A | Tumor necrosis factor receptor superfamily member 10A |
| TNFRSF11A | Tumor necrosis factor receptor superfamily member 11A |
| TNFRSF13B | Tumor necrosis factor receptor superfamily member 13B |
| TRAIL-R2 | TNF-related apoptosis-inducing ligand receptor 2 |
| VEGFD | Vascular endothelial growth factor D |
| VSIG2 | V-set and immunoglobulin domain-containing protein 2 |
| XCL1 | Lymphotactin |

References

1. Assarsson E, Lundberg M, Holmquist G, Björkesten J, Bucht Thorsen S, Ekman D, Eriksson A, Rennel Dickens E, Ohlsson S, Edfeldt G, Andersson A-C, Lindstedt P, Stenvang J, Gullberg M, Fredriksson S. Homogenous 96-Plex PEA Immunoassay Exhibiting High Sensitivity, Specificity, and Excellent Scalability. PloS one. 2014;9(4):e95192. doi:10.1371/journal.pone.0095192.
